# Medical versus social egg freezing: the importance of future choice for women’s decision-making

**DOI:** 10.1007/s40592-022-00153-9

**Published:** 2022-03-20

**Authors:** Michiel De Proost, Alexis Paton

**Affiliations:** 1 RHEA (Research Centre Gender, Diversity and Intersectionality), Vrije Universiteit Brussel, Brussels, Belgium; 2 Centre for Health and Society, Department of Sociology and Policy, Aston University, B4 7ET Birmingham, UK

**Keywords:** Empirical ethics, Oncofertility, Qualitative research, Respect for autonomy, Social egg freezing, Time

## Abstract

While the literature on oncofertility decision-making was central to the bioethics debate on social egg freezing when the practice emerged in the late 2000s, there has been little discussion juxtaposing the two forms of egg freezing since. This article offers a new perspective on this debate by comparing empirical qualitative data of two previously conducted studies on medical and social egg freezing. We re-analysed the interview data of the two studies and did a thematic analysis combined with interdisciplinary collaborative auditing for empirical ethics projects. Despite their different contexts, major similarities in women’s decision-making and reasoning were found. We developed two main common themes. Firstly, women felt a clear need to plan for future options. Secondly, they manipulated decision-times by postponing definitive decisions and making micro-decisions. The comparison highlights that the passage of time and the preservation of future choice seems to permeate all aspects of the patient experiences in both studies. As a result of considering real-world lived experiences, we suggest that there are many overlaps in women’s reasoning about egg freezing regardless of why they are making a decision to freeze. These overlaps are morally relevant and thus need to be further integrated into the existing arguments that have been canvassed in the flourishing egg freezing and fertility preservation debates across the field, and in policy and practice globally.

## Introduction

Advances in the last twenty years of assisted reproductive technology have made fertility preservation of gametes a real possibility for female patients whose reproductive function is threatened by treatments such as chemotherapy and radiotherapy, a field of medicine now called oncofertility (Woodruff et al. 2010; Woodruff, Shah, and Vitek 2019). The options available to preserve gametes for women has expanded from embryo freezing to include ovarian tissue freezing and medical egg freezing (MEF) (Argyle, Harper, and Davies 2016; Inhorn et al. 2018).

Success with these same freezing techniques has opened the possibility for healthy women to prevent future infertility. This new practice is called ‘social egg freezing’ (SEF), placing it in contrast to ‘medical’ uses such as in oncofertility (Mertes and Pennings 2011). Alternative terminology used to describe SEF, such as ‘planned oocyte cryopreservation’ (Daar et al. 2018), ‘oocyte cryopreservation for age-related fertility loss’, (ESHRE 2020) or ‘elective egg freezing’ (Inhorn et al. 2018a), show the ways this practice is judged differently to MEF.

This distinction has the moral implication that MEF is somehow more acceptable in most societies as it is perceived as disease-related and medically necessary, and thus conveying to these individuals a morally legitimate claim to access and use these services (Martin 2010). In some countries, MEF patients even receive these services for free, based on a justice argument (Kroløkke et al. 2019).

One of the main motivations for healthy women who choose SEF is that they lack a stable relationship or other life benchmarks they believe are important before having a child (de Groot et al. 2016; Kılıç and Göçmen 2018; Baldwin et al. 2019). SEF is considered by some as controversial because of its unnaturalness, the possible risks it can create for healthy women, and the biomedicalisation of a societal problem (Mertes 2018; Kostenzer, de Bont, and van Exel 2021; Petersen 2021). Despite its controversy, SEF has been described as one of the fastest-growing assisted reproductive technologies within Europe, the UK, and other parts of the world (Jackson 2018; Johnston et al. 2021).

Different authors have argued that the conceptual distinction between ‘social’ and ‘medical’ freezing is difficult to maintain and far from razor sharp (Petropanagos 2010; Dondorp et al. 2012; Pennings 2013; Petersen 2021; Rimon-Zarfaty et al. 2021). For instance, van de Wiel (2020) reasons that the seemingly common-sense opposition between ‘medical’ and ‘social’ freezing categorizes and polarizes a situation that is more complex than this binary suggests and is judging the use of SEF. Moreover, several scholars maintain that egg freezing is in important ways morally identical whether one undertakes it because of the likely sterilizing effects of cancer therapy or whether one simply anticipates delayed childbearing. Goold and Savulescu (2009) referred to the utilitarian principle of temporal neutrality, indicating that timing of the harm of infertility has no normative significance, to defend this position. Petropanagos (2010) argued that the broader social context of patriarchy shapes both choices in similar ways. For those authors it is more reasonable to respect the autonomy of women and allow egg freezing regardless from the underlying motivation.

While the literature on oncofertility decision-making was central to the bioethics debate on SEF when the practice emerged in the late 2000s (Goold and Savulescu 2009; Petropanagos 2010; Mertes and Pennings 2011), there has been little discussion juxtaposing the two forms of freezing since. Only a few qualitative studies examining SEF have included women who froze eggs for medical reasons, focusing on how women used this technology to reconcile different timelines (i.e., biological, social, and psychological) (Waldby 2015; Baldwin et al. 2019).

This article offers a new perspective on this bioethics debate by comparing empirical qualitative data on how women with cancer and healthy women who want to anticipate the risk of reproductive aging make fertility preservation decisions. It does so by presenting common themes developed from two previously conducted studies. This article will show how both groups of patients have the same active relationship with time and try to manipulate it as a way to deal with uncertainty and gain control, regardless of the reason why they wish to freeze eggs. By raising the issue of time, this study provides a new starting point to inform the debate on whether it is appropriate to maintain the existing demarcation between social and medical reasons for egg freezing in bioethics theory, and the policy and practice that theory informs.

## Methods

This article is based on the findings of two studies, each used here as a case study for exploring the importance of time in the decision-making process. These studies were designed independently of one another. The possibility that they might yield comparative insights for both patient groups was discovered after the data was collected. It was a ‘serendipitous discovery’ (Merton and Barber 2004) developed organically through discussion and re-analysis of this earlier collected material. This is not to ignore that independent oncofertility patients and social egg freezers constitute distinct social identities and realities; rather, it is to point to a connection between the two groups specific to their medical context.

The first study examined how British pre-menopausal women, diagnosed with cancer, made decisions about fertility preservation for medical reasons (Paton 2017; 2018; 2019). One-to-one, semi-structured interviews were conducted, prioritizing the lived experiences of those who participated in the study as the primary source of data. The interviews were structured so as to focus on the social, clinical and ethical concerns of the patients. Most of the women were told about both egg or embryo freezing by their doctors, but not all the women recalled being offered it, and some of the women were not aware that fertility preservation would have been possible before their cancer treatments. The study received ethical approval from Newcastle University and its associate NHS Trust (The Newcastle upon Tyne Hospitals NHS Foundation Trust).

The second study focused on women in Belgium who had considered or had undertaken at least one egg freezing cycle for so called social reasons (De Proost et al. 2021a; 2021a). Interviews were conducted based on a semi-structured questionnaire. At the beginning of the interview, open questions were asked to invite the participants to speak about SEF in their own words. In the second part of the interview participants were encouraged to reflect about ethical concerns related to SEF. The study was ethically approved by the Medical Ethics Committee of the University Hospital Brussels and Ghent.

To analyse and compare the interview data of the two studies we did a thematic analysis (Braun and Clarke 2006) combined with interdisciplinary collaborative auditing for empirical ethics projects (Provoost 2020). The latter method enabled a focus on the interdisciplinary challenges of our teamwork and balanced normative questions. Selections of raw data were analysed by both authors and possible themes and subthemes were discussed during auditing meetings. This process of revision was repeated until a consensus was reached and enhanced the rigor of our comparison.

Written consent was obtained from all participants before the interview in both studies. Pseudonyms are used throughout the paper and other personal information has been edited out. When quotes are presented, we provide descriptors to indicate the age and whether a participant had thought about fertility preservation for medical (M) or social (S) reasons.

## Results

The data of all participants, eleven interviews with medical freezers and twenty-one social freezers, were analysed. Two major themes relating to women’s decision-making of fertility preservation were identified when the results of both studies were compared: ‘Planning for the future’ and ‘Manipulating decision-times’.

### Planning for the future

Planning for the future was a key reason why fertility preservation was of interest to both sets of women. Regardless of why, women in both data sets expressed a need to plan for options in the future, which freezing offered them. Instead of having concrete plans for any potential frozen gametes, the women expressed a desire to provide for possible futures in which they could have the choice of a child once they had reached a period of stability and certainty.

Freezing was seen as one way to maintain control in an uncertain situation. Medical freezers expressed a frustration over what Paton (2018) and Scully et al. (2007) have both called ‘predictive fuzziness’ — an inability for doctors to quantify survival in concrete terms. Angela, who was not offered fertility preservation, but had wanted it, described the important role fertility preservation would have played in preserving not just her fertility, but mitigating uncertainty around her future choices:

‘If someone could’ve said we’re going to take something and keep it to one side […] Definitely, yeah. […] I would have said yes, keep the bit and then I can make my decisions later’ (Angela, 48, M).

Social freezers experienced a similar uncertainty resulting from predictive fuzziness due to different aspects of their life beyond personal control, such as the uncertainty in finding a partner and even the uncertainty of the medical procedures themselves being successful when the women were ready to use them. Erika described how there were still various factors outside the individual woman’s control in terms of anticipating infertility, like success rates:

It doesn’t guarantee success […] It’s not an exact science and it’s not because I’m going to freeze my eggs that I’m going to do IVF and that I’m going to be pregnant, so I think that’s a bit the duality in the story, it gives me a bit of a false form of reassurance or control. (Erika, 37, S)

Laura (31, S) reflected on the idea of control and described the process of social freezing as gaining ‘self-control’ over the situation, saying: ‘Because I actually want self-control over the rhythm in which I would develop my desire for children’. In both groups the need to control uncertainty, whether this was due to disease or other circumstances, created a perfect storm scenario in which freezing eggs was seen as one way to assert control and allay the unknown aspects of the women’s futures. Instead of waiting they could undertake action that preserved future choices. This resulted in a feeling for the women of being more in-control of an uncertain situation. Freezing was a form of preserving options in the future, allowing the women space to make important decisions about having children in their own time.

The ability to plan for the future also included the ability to plan contingencies in the event that natural conception was not possible. Different participants in both studies articulated clear planning motives, and spoke of fertility preservation as an alternative to the potential or inevitable failing of the reproductive body. The words ‘back-up plan’ or ‘plan b’ were often used to frame their intentions, as described by Jie (33, S): ‘The egg freezing is basically just a back-up plan. My estimation is to be able to conceive naturally in the next couple of years.’ Freezing offered a possible route to alleviating the uncertainty all the women felt by providing a contingency plan for fertility, such that several possible futures were secured for them.

Most participants actually hoped that they would not need to use their frozen eggs in the future, but the mere existence of the service served to reassure them that if the worst did happen and their primary plan of natural pregnancy did not occur, that the possibility of motherhood would not be lost. This was of particular importance where the women themselves were unsure of whether they would want children in the future, regardless of whether this was due to their cancer or other life circumstances. Several of the social freezers disclosed more ambivalent feelings towards motherhood:

Interviewer: Would you say that you already have a strong desire to have children?

Maaike: I think that’s a difficult question, not necessarily, not like other women around me or my age, no. In that long relationship I had that very strongly […] but at the moment I don’t really want to have a child. (Maaike, 35, S)

Ambivalence about motherhood was not confined to social freezers, with some medical freezers also feeling too young to have to make such grown up decisions. Stephanie (23, M), who was diagnosed with cancer right when she turned 18, felt she was far too young to make decisions about having children. As she put it: ‘At the time I wasn’t really bothered, you know. I’m only young and I’m not really bothered at the time. We’ll come to that when it comes to that.’ In similar vein, Monica, who was diagnosed with breast cancer as a young woman in her 20s, described the thought process that went into her decision:

And my oncologist at the time […] said that it might be worth giving it a go because I was only 24 and I hadn’t really given much thought to having children at that point. But I couldn’t say that I definitely didn’t want them […]. (Monica, 24, M)

The most important thing seemed to be to keep the option open. Egg freezing gave these women the opportunity to delay and actually suspend decision-making around motherhood until a later time when they became more confident in whether or not they wanted children, normally after reaching a milestone such as achieving their goal of finding the right partner or being cancer-free.

### Manipulating decision-times

A second major theme is what we describe as ‘manipulating decision-times’. Both groups manipulated the relationship between decision time and chronological time in several ways: some postponed definitive decisions to cope with their current situation, others anticipated future scenarios by making several incremental micro-decisions instead of only one concrete final decision.

For some participants, an aspect of their contingency planning was that they postponed decisions, placing them into a future, not to be dealt with in the present. This allowed the women to distance themselves from the decision no matter how urgent the decision may have been. For example, Jie stressed that, at the age of 33, motherhood was not really a priority:

So, to me having a plan is exactly the reason why I decided to do this: because I don’t have to think about this, because of the time I [still] have. […] So, I think I don’t have that concern for another couple of years. (Jie, 33, S)

For those women considering freezing for medical reasons, a similar hesitation to make decisions in the present led them to manipulate time by placing these same difficult decisions about having children into an unidentified future time. For example, Stephanie manipulated time to justify her decision not to make efforts to preserve her fertility during her treatment:

We’ll come to that when it comes to that. […] I don’t think until the time that I am wanting to have children will it really bother us that much. […] Obviously it will at the time if I do find out that I cannot. But I guess I don’t have to think about it at the minute, I’m too young. (Stephanie, 23, M)

She did express a desire to have children, but her concerns about whether she could seem to also exist in this undetermined future time when she was ‘wanting to have children’ and would be more able to cope with the prospect of having children.

The phrases ‘I don’t have to think about it at the minute’ (Stephanie, 23, M), and ‘I don’t have that concern for another couple of years’ (Jie, 33, S) were indicative of a style of time manipulation that highlighted expectations of and orientations towards the future. Similarly, Julie (34, S) noted that having children was such a ‘fundamental decision that you have to be ready for that’ and wanted to put off this important decision into a distant time that had yet to come, so it did not have to be dealt with in the present.

Another strategy was to anticipate future scenarios by making several micro-decisions. This was illustrated by Nina (33, S) when she revealed her decision-making process. Nina started considering egg freezing two years ago. During the first months she was thinking ‘it’s going to be okay, this is not necessary’ and made the micro-decision to do nothing. Then she said: ‘if nothing happens, I’m going to take action’. By her next birthday she met someone and thought ‘you see, it’s not necessary, it’s going to be all right’ but this relationship ended quickly. She needed some time to re-evaluate her previous decisions before she took up the egg freezing plan again. When we interviewed her, she was convinced of undertaking the procedure and declared that ‘it is now or never’. However, she admitted that feelings of doubt would come up again if she met someone in the coming months when the eggs are taken and frozen. This strategy was also reported by other social freezers highlighting how micro-decisions, related to the long-term goal of parenthood, were sequenced over time.

Similarly, the participants with cancer retrospectively described micro-decisions being made. For example, Monica’s first steps into considering fertility preservation were to reflect on her relationship at the time, which effectively ruled out embryo freezing as an option:

But at that time the guy that I was going out with, I definitely didn’t want to have children with. So when they were saying that we could have had embryos frozen and all that there was just no way that I wanted to do that […]. (Monica, 24, M)

As with all the other ways that the participants experienced and interacted with time, micro-decisions were a helpful way for participants to mitigate uncertainty, weigh up their options as best they could and help parcel out big weighty decisions into manageable ones that they could cope with.

## Discussion

To the best of our knowledge, this is the first study that explicitly compares empirical qualitative data of how both social and medical freezers make decisions about fertility preservation through the lens of empirical bioethics. Despite their different contexts, major similarities in women’s decision-making and reasoning were found. Both studies had two main common themes. Firstly, women felt a clear need to plan for future options. Secondly, they manipulated decision-times by postponing definitive decisions and making micro-decisions to exert control and mitigate uncertainty. The comparison highlights that the passage of time and the preservation of future choice seems to permeate all aspects of the patient experiences in both studies, regardless of why the women were considering fertility preservation.

The role of temporality is the common thread running through the women’s decision-making. Interestingly, this contextual factor is absent from most of the bioethics literature, though social sciences studies on SEF have highlighted its importance (Waldby 2015; Baldwin et al. 2019). Consistent with the latter literature our results demonstrate how a need for planning was an essential element in the narratives of both groups because of the significant uncertainty that a cancer diagnosis or other life circumstances can bring. Further, our study illustrates the ambivalence of some women toward motherhood even when they are actively freezing eggs (Myers 2017; Baldwin 2019). It seems a strategy to cope, handle and manage the affective and cognitive burden of difficult decisions about reproduction by prolonging decision time and slicing it up into manageable time intervals that were relevant to the women at the time.

Women in our study tried retaining control through two clear manipulation processes of time: (1) the postponing of definitive decisions and (2) micro-decisions. They balanced and compromised between the temporal arenas of their present and future selves. This accords with Scully et al. (2007: 217) earlier reflections on time and decision-making as a strategy ‘of reducing the temporal depth of field’ to preserve moral competence. In our results both groups seemed to manipulate time to create the moral space necessary to exercise their autonomy and make difficult decisions they felt comfortable with and that accorded with their needs, values, and beliefs.

Previous bioethics literature has suggested that the ethical dichotomy between MEF and SEF is not straightforward and is likely not a dichotomy at all (Goold and Savulescu 2009; Petropanagos 2010; Mertes and Pennings 2011; Rimon-Zarfaty et al. 2021). Our comparison provides new empirical evidence that reinforces this normative claim, showing that it is not just a theoretical possibility, but is the practical reality of how women make decisions about fertility preservation. Rather than playing medical freezing against social freezing, it may be more meaningful to understand these practices as interwoven, especially if we take these temporal experiences into account. There seems to be many overlaps between the two studied groups of women, as the preservation of future choice for the sake of getting more time, creates morally analogous reasons for fertility preservation. Our empirical data indicates that the actual reasoning in each scenario is parallel and less different than has previously been argued in the literature. Argumentations that start from a strict arbitrariness on which form of freezing is ‘right’, ‘allowed’ or ‘justified’ are missing the mark in understanding these similarities and do not reflect women’s actual practice.

When it comes to the concept of autonomy, a particular thin conception of autonomy is often played out in the bioethics literature of egg freezing, where it is understood as retaining individual choice and control over the planning of pregnancy (Goold and Savulescu 2009). A more diachronic and relational understanding of autonomous choice seems necessary (Baumann 2008; Wardrope 2015; Paton 2018; Childress 2022), to better reflect on how present actions may affect futurity, but also simply to reflect the reality of how patients make decisions. However, some relational conceptions of autonomy may be tempted to go so far as to say these women may have been struggling to exercise their autonomous choice as they wrestled with uncertainties and lack of control—though we disagree. As one of the authors argued in a previous paper, women ‘use time to assert their autonomy by creating space in which to make decisions, or “keep” decisions until a later time’ (Paton 2018: 83).

While it is beyond the scope of this paper to evaluate all the normative implications of our empirical data, for instance whether or not egg freezing should be covered within the healthcare system and related considerations of broader access and social justice (Campo-Engelstein 2010; Mertes and Pennings 2012; Johnston et al. 2022), a new round of policy debate is necessary in light of the similarities of experience of both groups of women. Further empirical and normative work is now needed to fully capture and evaluate different stakeholders’ viewpoints on the medical/non-medical distinction used in the allocation of egg freezing funding.

## Limitations

Our comparison approach carries some limitations. Firstly, the findings are based on two different country contexts, the United Kingdom and Belgium, and cannot be generalised to all women undergoing egg freezing across all countries. Secondly, the research designs were not developed for the purpose of being compared, therefore participants were recruited and interviewed differently, though the studies were independently designed with the same methodological approach, which we feel mitigates this limitation. A study which purposively includes both groups of egg freezers would explore the discussed issues in greater depth, and is in development by the authors.

## Conclusions

The leitmotif throughout this research was the importance of future choice as a contextual element in reproductive decision-making in the field of fertility preservation. Two major themes were found by comparing the data of previous conducted studies on MEF and SEF: ‘Planning for the future’ and ‘Manipulating decision-times’. For both groups of patients, the ability to have a future choice was central instead of making an instant choice that would mortgage their future options. As a result of considering real-world lived experiences, we suggest that there are many overlaps in women’s reasoning about egg freezing, regardless of why they are making a decision to freeze. These overlaps are morally relevant and thus need to be further integrated into the existing arguments that have been canvassed in the flourishing egg freezing and fertility preservation debates across the field, and in policy and practice globally.
